# Network robustness assessed within a dual connectivity framework: joint dynamics of the Active and Idle Networks

**DOI:** 10.1038/s41598-017-08714-3

**Published:** 2017-08-17

**Authors:** Alejandro Tejedor, Anthony Longjas, Ilya Zaliapin, Samuel Ambroj, Efi Foufoula-Georgiou

**Affiliations:** 1 Department of Civil and Environmental Engineering, University of California, Irvine, CA USA; 2 Department of Mathematics and Statistics, University of Nevada, Reno, NV USA; 3 Steinbuch Centre for Computing, Karlsruhe Institute of Technology (KIT), Karlsruhe, Germany

## Abstract

Network robustness against attacks has been widely studied in fields as diverse as the Internet, power grids and human societies. But current definition of robustness is only accounting for half of the story: the connectivity of the nodes unaffected by the attack. Here we propose a new framework to assess network robustness, wherein the connectivity of the affected nodes is also taken into consideration, acknowledging that it plays a crucial role in properly evaluating the overall network robustness in terms of its future recovery from the attack. Specifically, we propose a dual perspective approach wherein at any instant in the network evolution under attack, two distinct networks are defined: (i) the Active Network (AN) composed of the unaffected nodes and (ii) the Idle Network (IN) composed of the affected nodes. The proposed robustness metric considers both the efficiency of destroying the AN and that of building-up the IN. We show, via analysis of well-known prototype networks and real world data, that trade-offs between the efficiency of Active and Idle Network dynamics give rise to surprising robustness crossovers and re-rankings, which can have significant implications for decision making.

## Introduction

Recent developments in understanding the structure and dynamics of networks have transformed research in many fields, ranging from protein interactions in a cell to page connectivity in the World Wide Web, to landscape drainage patterns and relationships in human societies^1–10^. Although these complex networks have different evolution rules, many exhibit a universal scale-free topology wherein the highly-connected nodes, although sparse, dominate the connectivity of the network^2^. Network robustness is commonly defined as the capacity of the network to maintain functionality (or connectivity) when a sequential node removal strategy (attack) is performed. Attacks can encode the action of very diverse processes acting on a network (ranging from actions of external agents to competing processes within the network) that result in binary outcomes bringing *active* nodes to an *idle* state. The robustness of networks with different topologies to different attacks has been widely studied, and different strategies to manage perturbation spread within the network have been suggested^3–6, 11–30^. Those studies have proposed a wide spectrum of methodologies and metrics to quantify the robustness of the networks, albeit focusing mainly on the connectivity of the nodes unaffected (Active Network) by the attack, while the connectivity of the affected nodes (Idle Network) has received minimal attention.

In this paper, we present a rationale behind the necessity of considering the connectivity of the Idle Network to suitably assess network robustness. To do this, we answer three basic questions. (1) Is it important to know the connectivity of the Idle Network? (2) Can the evolving properties of the Idle Network be inferred from those of the Active Network alone? (3) How sensitive is the assessment of robustness to the structure of the Idle Network?

## Is it important to know the connectivity of the Idle Network?

We motivate, via three examples, the necessity of using a dual perspective approach to assess robustness, where the connectivity of the Idle Network is also considered.

- *Stabilization of damage*. The dynamics of competing species, e.g., native vs. invasive, in an ecosystem can be also modeled as an attack. Notwithstanding that the robustness (e.g., survival capacity) of native species requires connectivity, connected loci dominated by the invasive species (Idle Network) can stabilize their population and hence diminish the overall long term robustness of the native community (e.g., native mussel vs. zebra mussels^31^).
- *Community Structures*. Many networks exhibit community structures, wherein nodes within the same community have higher connectivity among themselves than with nodes outside of their community^32^ (e.g., employees within a department). This characteristic topology often responds to the specialized functionality and dynamics of those communities (e.g., different departments in a company), and therefore the disconnection of a significant number of within-community nodes (Idle Network) can affect the functionality of the whole network, even if the overall connectivity of the network is not significantly affected (e.g., the effect of the same number of people absent in a company can instigate a much larger decline in network functionality when they belong to the same department–highly connected nodes–since the specialized function assigned to their department can be compromised, jeopardizing the functionality of the whole system).
- *Cascading Failures*. In power grids, after the failure of a node or a link, the electric currents are instantaneously diverted to neighboring active nodes due to the impossibility of storage. The subsequent failure of formerly active nodes (Idle Network) that were connected in a locality can provoke excess current in the surrounding nodes that might lead to cascading failures in the whole network (e.g., 1996 blackout originated in Oregon, which affected eleven U.S. states and two Canadian provinces^33^).

These three examples demonstrate that the connectivity of the Idle Network can be crucial in assessing the overall network robustness. However, a fair question can be raised at this point: Since the Active and Idle Networks both emerge from the action of the same attack on a network, can the information contained in the Idle Network be inferred from the connectivity in the Active Network? We address this question in the next section.

## Can the evolving properties of the Idle Network be inferred from those of the Active Network alone?

To answer this question let us define formally both the Active and Idle Networks. Consider a network *N* that consists of nodes {*n*_*i*_}, *i* = 1, …, *T* connected by edges {(*n*_*i*_, *n*_*j*_)} (here considered undirected). We focus on a process of *sequential node removal*, also called an *attack*. The process starts at *t* = 0 with the original network *N*. At each discrete time step *t* > 0 it eliminates a suitably chosen node *n*_i_ and all edges (*n*_*i*_, **·**) connected to this node, resulting in the set of nodes and edges, called the Active Network *N*_*A*_(*t*), that have been unaffected and thus are active at *t*. This sequential node removal operation can mimic a multitude of actual processes acting on networks and having a binary outcome, e.g., healthy species in a biological community that may become sick, clean streams in a river network that may become contaminated, people that may learn particular information, *etc*. We also consider the Idle Network *N*_*I*_(*t*) that consists of the nodes that have been removed from *N* up to time *t*, together with all the edges from *N* among these idle nodes. Accordingly, a sequential node removal process *D* results in the following decomposition of the network *N*:

1 $$D:N\to \{{N}_{A}(t),{N}_{I}(t)\},t=1,\ldots ,T.$$

Observe that the union of the nodes in the *N*_*A*_(*t*) and *N*_*I*_(*t)* networks matches the set of nodes in the original network *N*. At the same time, the union of edges from *N*_*A*_(*t*) and *N*_*I*_(*t)* is only a subset of the edges in the original network, since the latter may also include some edges that bridge across the evolving *N*_*A*_(*t*) and *N*_*I*_(*t*) networks. In other words, the pair {*N*_*A*_(*t*), *N*_*I*_(*t*)} cannot be used in general to reconstruct *N*; although *N*_*A*_(*t*) is uniquely determined by {*N*, *N*_*I*_(*t*)} and *N*_*I*_(*t*) is uniquely determined by {*N*, *N*_*A*_(*t*)}.

**Figure 1:**
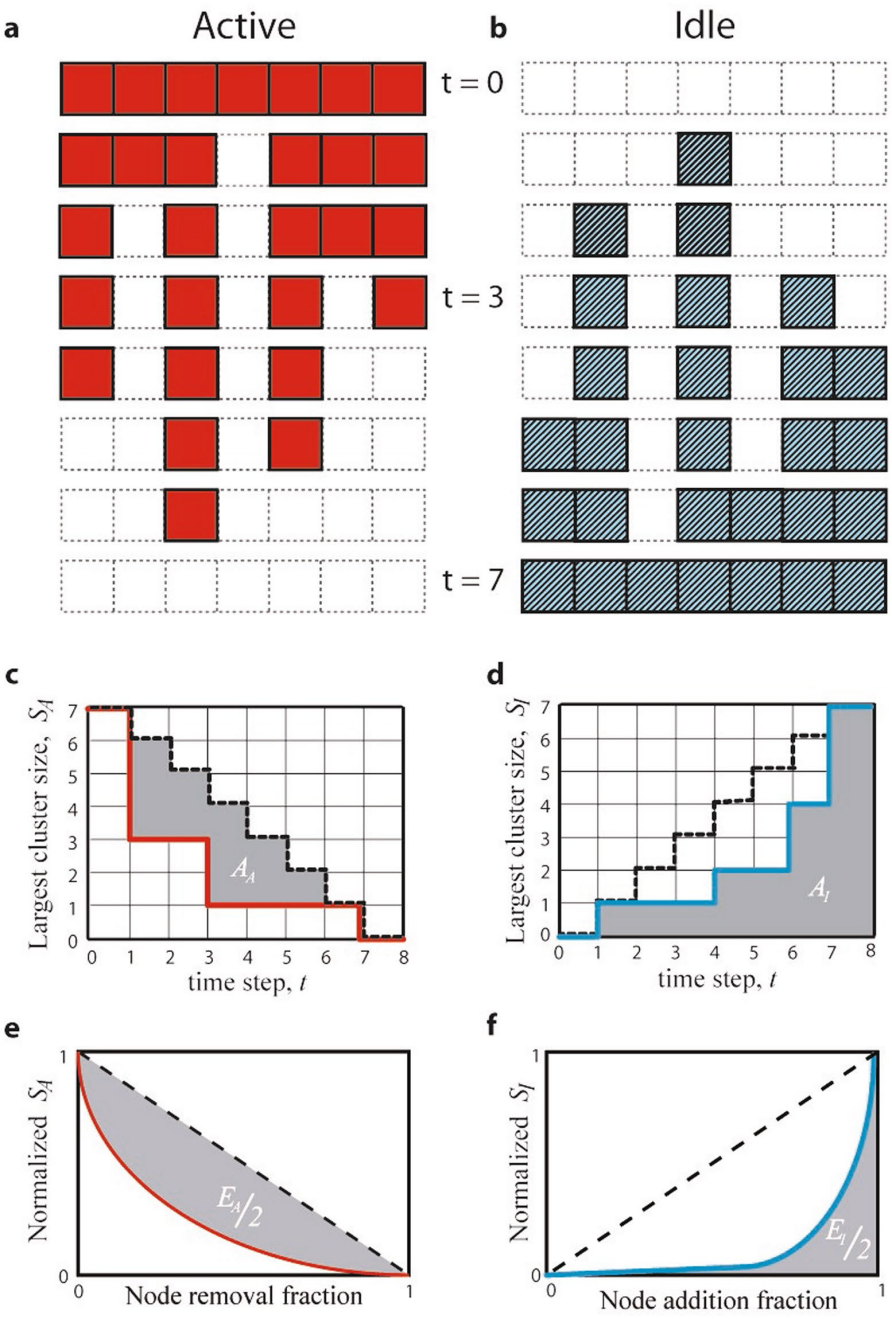
Dual connectivity perspective in a simple line network. (**a**) At time *t* = 0 the line network consists of seven nodes, all belonging to the Active Network (AN) shown as solid squares. At each time step, one node is removed to destroy the connectivity of the AN in the most efficient way. (**b**) Each removed node in the AN creates a node in the Idle Network (IN) shown as stripped squares. The largest cluster size *S*_*A*_ (*S*_*I*_) in the AN (IN) is shown by a solid line in panels (**c**) and (**d**). It is observed that *S*_*A*_ and *S*_*I*_, evolve asymmetrically: the most efficient procedure to reduce *S*_*A*_ is not the most efficient to increase *S*_*I*_. The efficiency of an attack has two components, *E*_*A*_ and *E*_*I*_, one for each perspective, and their values are proportional to the gray area in panels (**e**) and (**f**) respectively. This illustrates that defining robustness in terms of only efficiency *E*_*A*_ or in terms of both efficiencies *E*_*A*_ and *E*_*I*_ could make a significant difference in assessing the overall system robustness.

We assert that the dynamics of *N*_*A*_(*t*) and *N*_*I*_(*t*) are not trivially related and therefore, a robustness metric of the network *N* should consider both of them. We illustrate the importance of this dual perspective by considering an example of node removal in a simple line-connected network of length *T* = 7 shown in Fig. 1. The connectivity of the network is assessed here by the size *S*(*t*) of its largest cluster; this is a conventional metric used in many previous studies^5, 11–30^. We implemented a strategy of node removal that is the most efficient in decreasing the size *S*_*A*_(*t*) of the maximal cluster of the AN (Fig. 1a). During the first three time steps the max cluster size decreased from 7 to 1. However, this particular strategy of node removal is not at all efficient with respect to building-up the connectivity of the IN (Fig. 1b): in the first three time steps the maximum cluster size *S*_*I*_(*t*) merely increased from 0 to 1.

Quantitatively, the *efficiency E*_*A*_ of a node removal strategy in destroying the AN can be defined as:

2 $${E}_{A}=\frac{{A}_{A}}{{A}_{max}}=\frac{{\sum }_{t=0}^{T}(T-t-{S}_{A}(t))}{{\sum }_{t=0}^{T}(T-t)}=1-\frac{2}{T(T+1)}\sum _{t=0}^{T}{S}_{A}(t)$$

here *A*_*A*_ is the area between *S*_*A*_(*t*) and the diagonal staircase (*T* − *t*) as in Fig. 1c and *A*_max_ is the area below the diagonal staircase. Similarly, the efficiency *E*_I_ of building the Idle Network can be defined as (Fig. 1d):

3 $${E}_{I}=\frac{{A}_{I}}{{A}_{max}}=\frac{{\sum }_{t=0}^{T}{S}_{I}(t)}{{\sum }_{t=0}^{T}(T-t)}=\frac{2}{T(T+1)}\sum _{t=0}^{T}{S}_{I}(t)$$

where *A*_*I*_ is the area below the curve *S*_*I*_(*t*). Obviously *E*_*A*_ ≠ *E*_*I*_ highlighting that the evolution of the connectivities in the Active and Idle networks are distinct.

**Figure 2:**
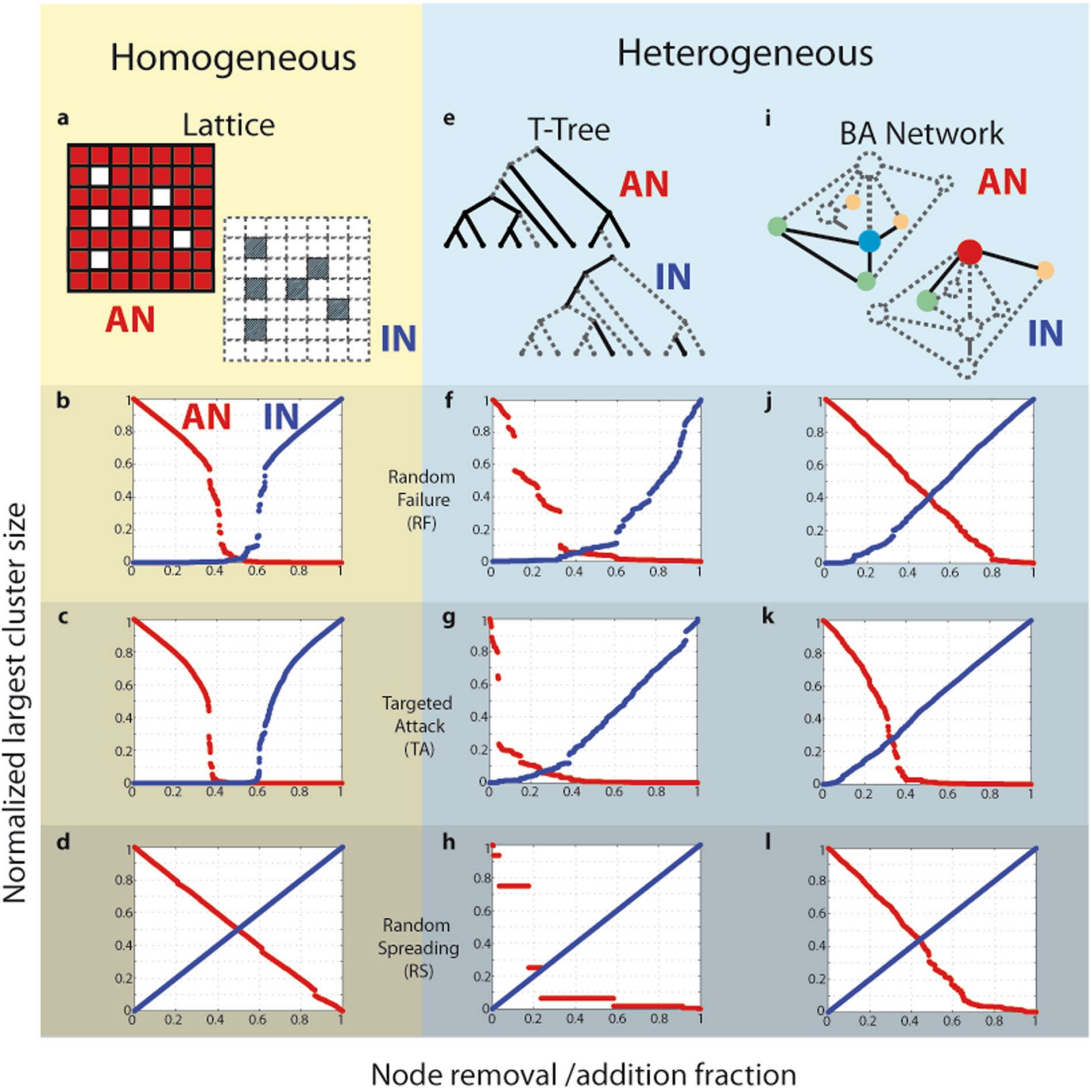
Dual connectivity perspective evolution of networks under attack. Evolution of the largest cluster size in the Active Network, AN (red) and Idle Network, IN (blue) for homogeneous (yellow panels) and heterogeneous (blue panels) networks with respect to three different sequential node removal strategies: panels (**b**,**f**,**j**) random failure, (**c**,**g**,**k**) targeted attack, and (**d**,**h**,**l**) random spreading. The largest cluster size and time are normalized by the system size. Three main observations are made: (i) the rate of decrease of the largest cluster size in the AN is not the same as the rate of increase of the largest cluster size in IN (asymmetric evolution); (ii) for homogenous networks and networks under random failure, there is a symmetry with respect to the vertical axis at 0.5 implying a complementarity in the efficiencies of destroying AN and building-up IN, i.e., *E*_*A*_ + *E*_*I*_ ≈ 1; and (iii) for heterogeneous networks (T-Trees and BA networks) and heterogeneous attacks (TA and RS) no symmetry is observed at all; there is a necessity to monitor both networks (AN and IN) since it is not possible to predict the value of the efficiency of building-up the IN from the efficiency value of destroying the AN and vice versa.

Below we examine the proposed dual perspective connectivity approach when three different strategies of node elimination (attack strategies) are applied to three widely used types of networks. Specifically, the networks that we employ in our analysis are: Network 1–A *square lattice* of *T* = 10,000 nodes arranged in a Von Neumann neighborhood (i.e., each node having four neighbors); see Fig. 2a; Network 2–A *Tokunaga self-similar tree* ^34^ (T-tree) of order Ω = 6, where each Horton-Strahler branch^7, 35^ in the tree represents a node, and parameters (*a*, *c*) = (1, 2) (see Fig. 2e); Network-3: A Barabasi-Albert (BA) *scale-free network* ^2^, with parameters (*m*_0_, *m*) = (3, 2) and *T* = 1,000 nodes (Fig. 2i). For further details about the examined networks see Methods. The aforementioned networks are classified according to the node degree distribution into *homogeneous* (lattice) and *heterogeneous* (T-tree and BA network).

In each system, we examine three strategies of node removal. Strategy 1- A *random failure* (RF) removes nodes at random using a discrete uniform distribution over all the active nodes. Strategy 2- A *targeted attack* (TA) assigns a removal probability to a vertex proportional to its degree of connectivity in the AN. Strategy 3- A *random spreading* (RS) removes the first node at random as in RF; afterwards, at each time step one node connected to an eliminated node is randomly removed. The evolution of the largest cluster size *S* under progressive node removal is examined using 100 simulations. Figure 2 shows *S*(*t*) as a function of time in the AN and IN for a representative realization from all the simulations for each network and attack; the time *t* is normalized to be equal to the fraction of the removed nodes. The first observation is that the rate of increase of the largest cluster size in the IN is not the same, in general, as the rate of decay of the largest cluster size in the AN. A lattice network under RS is an exception–the symmetry here (with respect to *S(t)* = 0.5) is expected by construction and it can only be altered by abrupt jumps in *S(t)* due to finite size effects. We also notice symmetry of *S*_*A*_ *(t)* and *S*_*I*_ *(t)* with respect to the vertical axis *t* = 0.5 that is only observed for a homogeneous lattice network (under any attack) and random failure (applied to any network) and as a consequence *E*_*A*_ + *E*_*I*_ ≈ 1 (see Table 1). The symmetry is not obvious in Fig. 2f due to the large jumps of the largest cluster size; although it can be shown statistically via the efficiency values (Table 1). Having the complementary values of *E*_*A*_ and *E*_*I*_ has an obvious but important implication: the more efficient a strategy is according to one perspective (e.g., destroying the connectivity in the AN), the less efficient it is according to the other (e.g., building-up the connectivity in the IN). Another important observation is that for T-trees, the connectivity of the AN is destroyed faster, and the connectivity of the IN is built up slower, than in the BA network. Finally, the perfect efficiency of the random spreading in the Idle Network is a consequence of its definition (*S*_*I*_ grows linearly).

**Table 1:** Efficiencies for the three attack strategies applied to the Lattice, Tokunaga Tree (T-Tree) and Barabasi Albert (BA) networks.

Attack	Lattice	T-Tree	BA Network
Random Failure (RF)	***E***_***A***_ = **0.35** ± **0.01** ***E***_***I***_ = **0.65** ± **0.01**	***E***_***A***_ = **0.58** ± **0.10** ***E***_***I***_ = **0.39** ± **0.10**	***E***_***A***_ = **0.17** ± **0.02** ***E***_***I***_ = **0.83** ± **0.02**
Targeted Attack (TA)	***E***_***A***_ = **0.42** ± **0.00** ***E***_***I***_ = **0.58** ± **0.01**	*E*_*A*_ = 0.87 ± 0.04 *E*_*I*_ = 0.75 ± 0.04	*E*_*A*_ = 0.48 ± 0.02 *E*_*I*_ = 0.94 ± 0.01
Random Spreading (RS)	***E***_***A***_ = **0.02** ± **0.02** ***E***_***I***_ = **1**	*E*_*A*_ = 0.76 ± 0.09 *E*_*I*_ = 1	*E*_*A*_ = 0.19 ± 0.02 *E*_*I*_ = 1

*E*_*A*_(*E*_*I*_) is the efficiency of an attack strategy in destroying (building) the Active (Idle) network. Values in bold represent complementary efficiencies (*E*_*A*_ + *E*_*I*_ ≈ 1).

Although the size of the largest cluster is a standard metric in network robustness studies^5, 11–30^, other statistics have been proposed to monitor the decay of the connectivity of networks under attack^11, 15, 18, 20, 36–43^. Each of these metrics have certain limitations in their assessment of network robustness, for instance, the evolution of the largest cluster size can be insensitive to attack strategies that sparsify networks without fragmenting the largest component. In such cases, the size of the largest cluster would decrease linearly with time concealing important transitions in the system connectivity. The proposed dual perspective approach is not limited to the utilization of the largest cluster size as a proxy for connectivity. Symmetries (and asymmetries) similar to those reported in Fig. 2 are observed with other metrics such as the diameter^11, 18, 36^, inverse geodesic distance^15, 37–40^, average cluster size^11, 18, 20^ and number of clusters^41–43^ (see Figs S1–S4 in the Supplementary Information).

The simultaneous analysis of the evolution of connectivity in the AN (decaying) and in the IN (building-up) reveals critical information that is not attainable from the analysis of the AN alone, calling for the development of a new framework to assess network robustness.

## How sensitive is the assessment of robustness to the structure of the Idle Network?

We propose a dual perspective network robustness metric *R*_*N*_ as a function of both the efficiency *E*_*A*_ of destroying the connectivity of the AN and efficiency *E*_*I*_ of building-up the connectivity of the IN:

4 $${R}_{N}=f({E}_{A},{E}_{I})$$

For illustrative purposes and in the absence of specific reasons for non-linearity of the function *f*(*E*_*A*_, *E*_*I*_), a simple metric of network robustness would be comprised of a weighted average of the two efficiencies, i.e.,

5 $${R}_{N}(\alpha )=\,\alpha (1-{E}_{A})+(1-{\alpha })\,(1-{E}_{I})$$

where *α* is the weight given to the efficiency of the AN while (1 − *α*) is the complementary weight given to that of the IN. Using *α* = 1 leads to a particular definition that is currently used in the literature to guide, for example, decisions on most effective strategy of attack or to assess recovery rates under a given attack^5, 11–30, 36–43^. While this may be a good approximation for some systems, it is restrictive for many others as we discussed in section 1. Thus, *α* < 1 values are needed to capture possible trade-offs on the relative importance of the connectivity of the AN and IN in assessing the overall system robustness to the attack.

**Figure 3:**
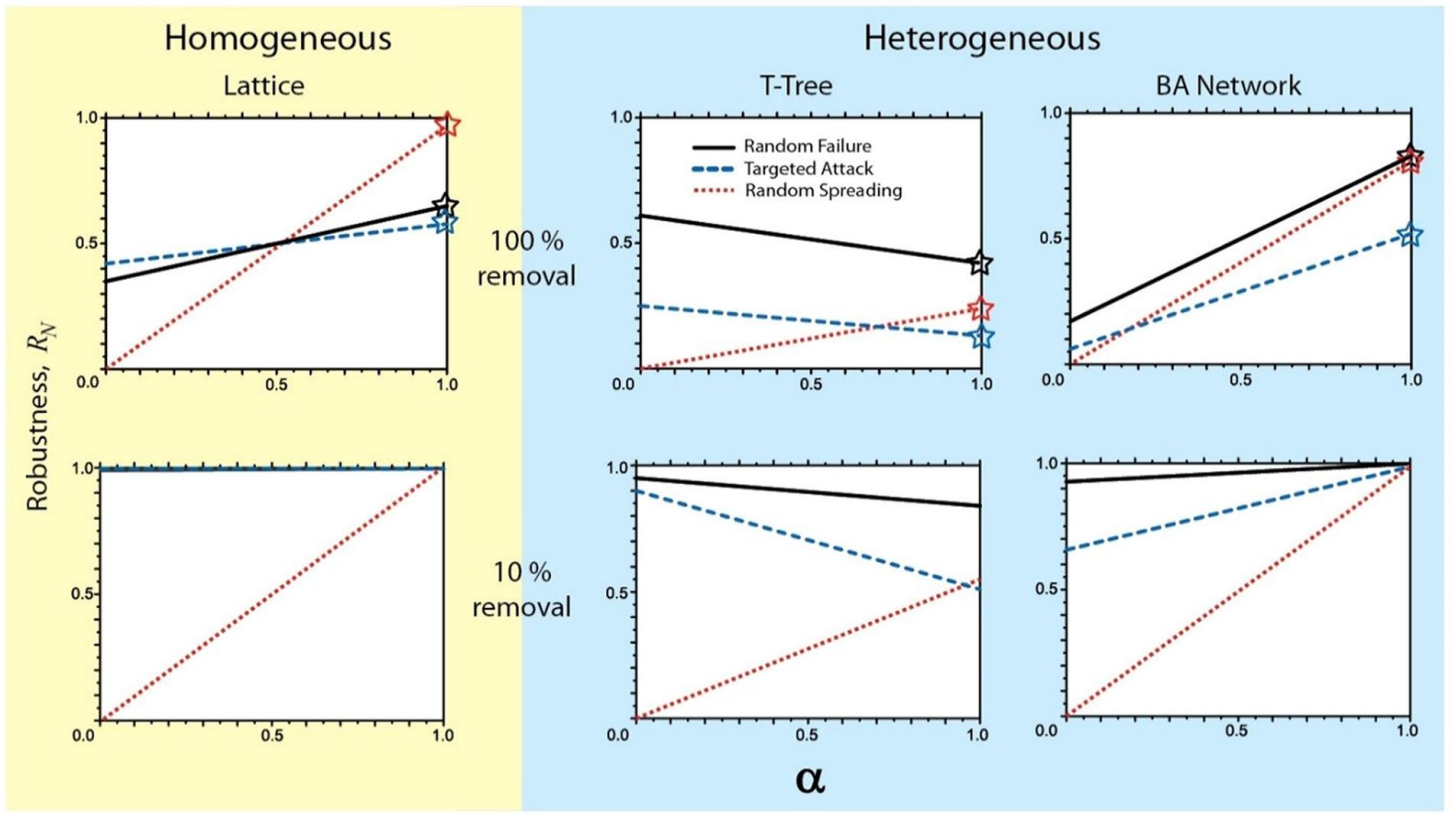
Robustness, *R*_*N*_, as a function of the relative weight given to the connectivity of the Active Network (AN), *α*. The robustness defined exclusively in terms of the AN (*α* = 1) is shown by stars. For the top panels, the robustness of a homogeneous network subject to any attack and heterogeneous networks under random failure, is equal to 0.5 for *α* = 0.5 due to the property *E*_*A*_ + *E*_*I*_ ≈ 1. For all cases, notice (i) a strong *dependence* of robustness on *α*, (ii) *robustness crossovers*–changes in ranking (ordering of respective *R*_*N*_ values) of different attack strategies depending on *α* and (iii) shift of the *robustness crossovers* towards *α* = 1 with substantial divergence in the attack strategies when the system is evaluated not at the time of complete destruction but at its early stages of attack (bottom panels).

To illustrate the consequences of implementing this dual perspective framework for network robustness assessment, we show in Fig. 3 (top panels) the values of network robustness (as defined in equation 5) as a function of the parameter *α* for the three different networks and attacks analyzed in section 2. Notably, the robustness may deviate substantially from the case *α* = 1 (marked by stars in Fig. 3), which is examined in most of the existing studies^5, 11–30, 36–43^. It is worth noting that our definition of robustness (equation 5) not only gives different numerical values, but also may result in *crossovers*–alternative ranking of attack strategies depending on the value of *α*. For example, in a lattice network, a crossover occurs at *α* = 0.5, with *R*_*N*,*RS*_ > *R*_*N*,*RF*_ > *R*_*N*,*TA*_ for *α* > 0.5, and *R*_*N*,*TA*_ > *R*_*N*,*RF*_ > *R*_*N*,*RS*_ for *α* < 0.5 (here the second lower index refers to the attack type). A crossover between *R*_*N*,*TA*_ and *R*_*N*,*RS*_ is also observed for T-trees at *α* ≈ 0.68 as well as for the BA network at *α* ≈ 0.17. Hence, an interplay between the AN and IN introduces a whole new dimension in the study of robustness, which cannot be reproduced by exclusively examining the AN. At the same time, some general observations remain consistent with previous works when *α* = 1, in particular those showing that networks are more robust under random failure than targeted attack. Other observations for *α* = 1 are: (1) for both the heterogeneous networks, *R*_*N*_ is highest for random failure, followed by random spreading and targeted attack; (2) the robustness in homogeneous networks is highest for random spreading, followed by random failure and targeted attack; (3) the *R*_*N*_ *-*value for random spreading and homogeneous networks is approximately equal to 1 since *S*_*I*_ grows linearly by definition and the efficiencies are complementary (*E*_*A*_ = 0, *E*_*I*_ = 1).

The results presented so far considered that the same node removal rules (time-invariant attack) operated on the system until its complete destruction. In many systems however, an adaptive “attack and recovery strategy” is applied, i.e., system performance is evaluated periodically, and especially in the early stages of the attack, to guide future actions. It is understood, for example, that an attack strategy, which is optimal when evaluated over a long period of time might be suboptimal relative to a shorter time horizon. Figure 3 (bottom panels) shows the results of the robustness-based ranking of attack strategies defined with respect to a partial (10%) system destruction. Although both the strong dependence of robustness on α and the presence of crossovers is still observed, the crossover location moves closer to *α* = 1 with substantial divergence in the attack strategy rankings for *α* < 1. The practical implications of this finding can be substantial; for example, in a BA network *α* = 0.7 (which gives 70% weight to the AN and 30% to the IN) would remarkably re-rank the robustness of different attack strategies which for *α* = 1 would be indistinguishable (rightmost bottom panel plot of Fig. 3).

**Figure 4:**
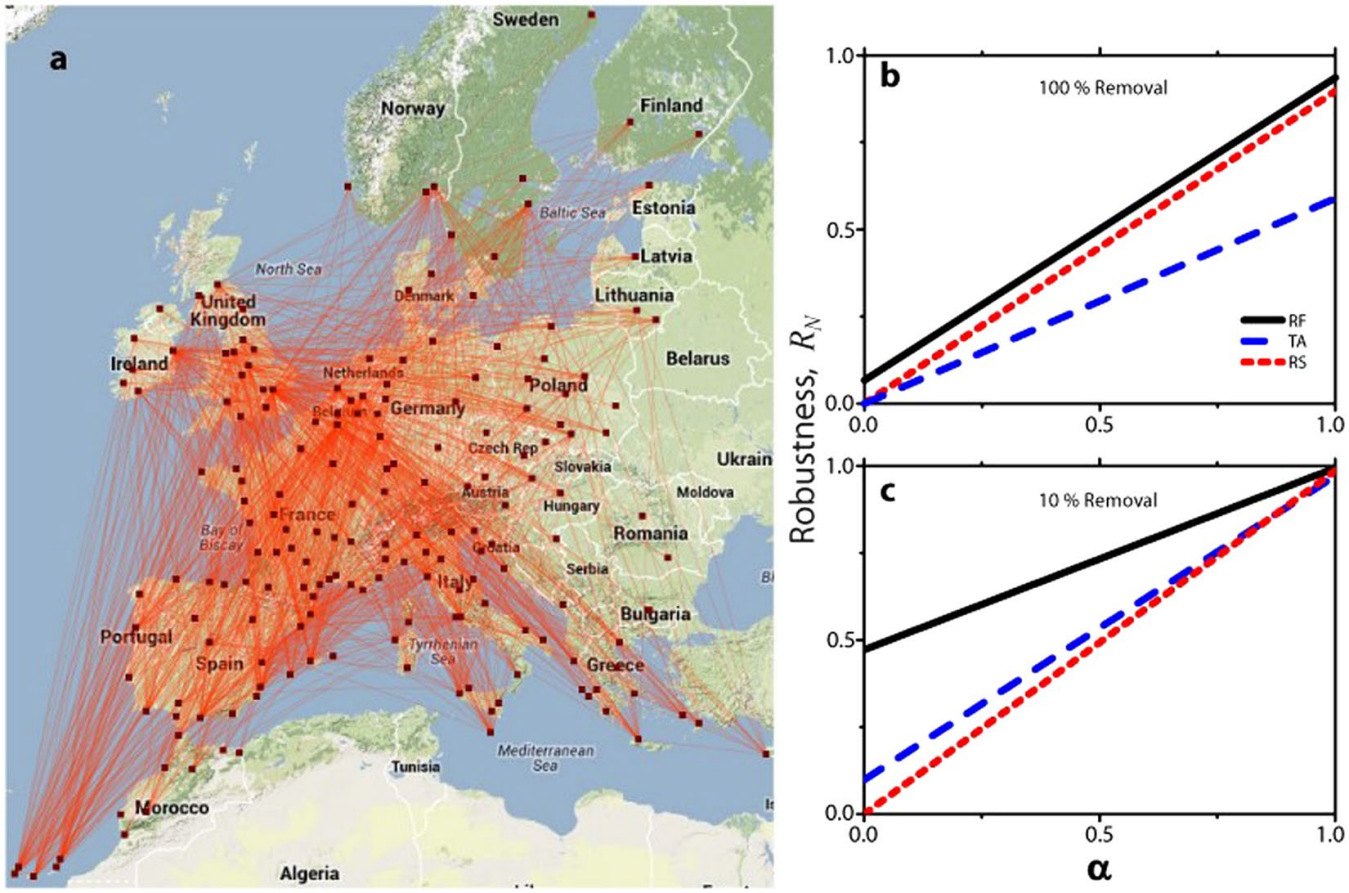
Robustness, *R*_*N*_, of the second largest airline network in Europe. (**a**) The map shows the connectivity of the second largest airline in Europe (RyanAir), operating in 186 airports (black squares), with more than 1500 routes (red lines). The map was generated using the *Google Maps API* and *Map Data ©2014 Google*. (**b**) shows the robustness of that network under three different attacks (Random Failure (RF), Targeted Attack (TA), and Random Spreading (RS)), which act until all the airports are removed. In panel (**c**), the robustness is evaluated under partial attack (10 % airports removed). Note that for *α* = 1, the network is equally robust under any of the three attacks. However, for *α* < 1 the robustness values for different attack strategies significantly differ, highlighting the importance of the Idle Network in assessing the robustness of the system.

To further illustrate the importance of the Idle Network in assessing system robustness, we consider data from the second largest European airline, RyanAir^44^. The examined network consists of 186 airports and 1507 edges that represent the existence of at least one weekly flight between the respective airports (Fig. 4a). Figure 4b shows the robustness values for a sequential removal of airports until all of them are inoperative (100% removal), according to the three previously implemented attack strategies: RF, TA and RS. The results are consistent with our simulations: (1) Random Failure generates complementary efficiencies (*E*_*A*_ + *E*_*I*_ ≈ 1). (2) Robustness ranking for the different strategies is similar to the BA network (see Fig. 3) and the crossover between TA and RS is observed near *α* = 0. Figure 4c shows the network robustness when the attack strategies act only until 19 airports are removed (10% node removal). Qualitatively, we have the same behavior as in the BA network (c.f. Fig. 3). However there exist significant quantitative differences, expressed in much lower values of robustness for *α* < 1. This is due to the structure of the airline network (point-to-point), which has numerous connections among all the airports and hence relatively high connectivity degree for all nodes, not only the hubs. Thus, it is more likely to build-up clusters in the IN than hub-and-spoke scale-free networks.

**Figure 5:**
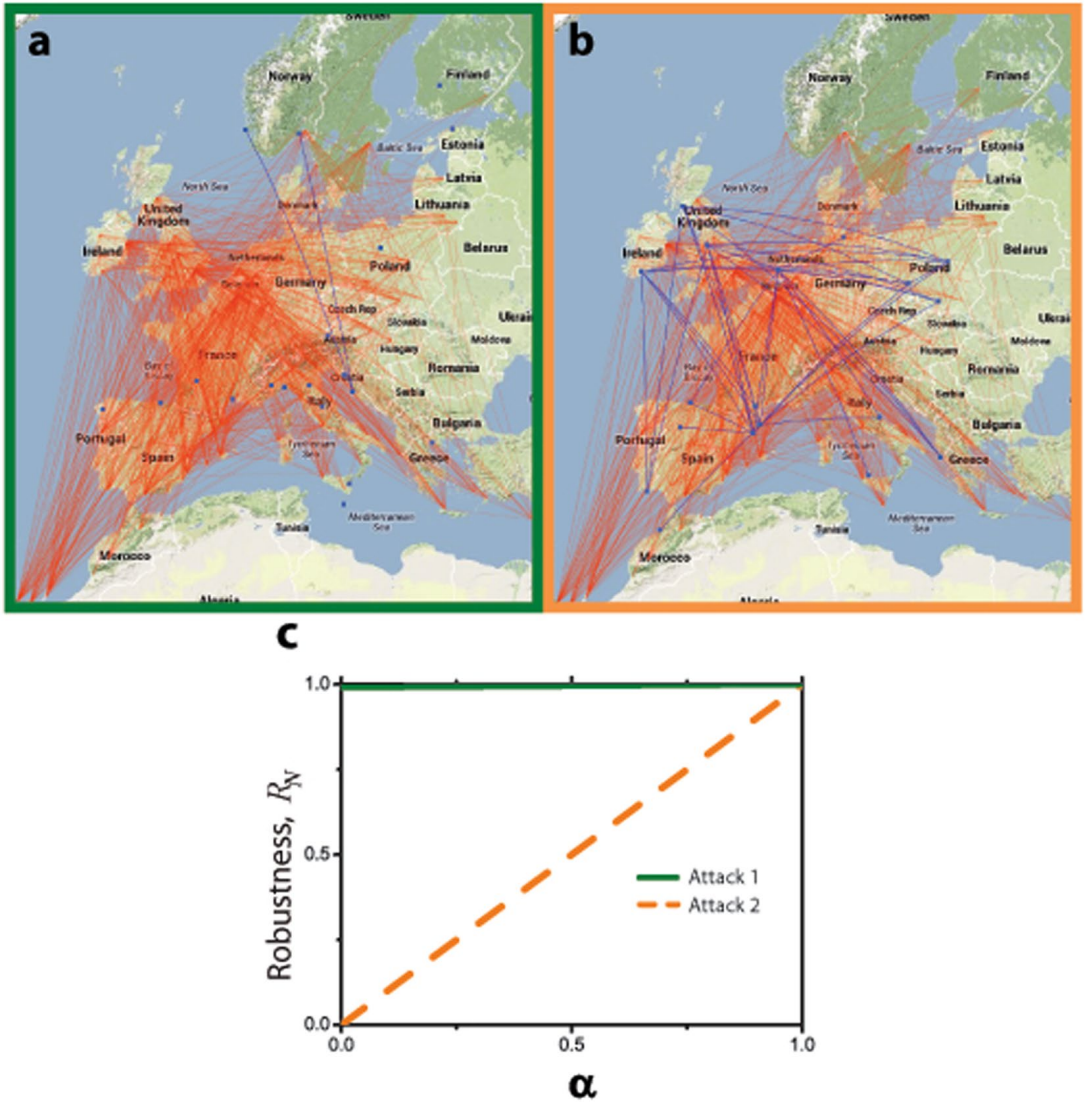
Importance of the dual connectivity perspective framework in assessing network robustness. The maps illustrate the result of two different attacks, Attack 1 and Attack 2, applied to the network until 19 airports are disconnected (10% removal). The two resulting networks have the same largest cluster size in the Active Network (*S*_*A*_ = 167), but different largest cluster size in the Idle Network: (**a**) *S*_*I*_ = 3 for Attack 1 and (**b**) *S*_*I*_ = 19 for Attack 2. Considering connectivity of the Idle Network in assessing the system robustness reveals significant differences in these two attacks, as quantified in (**c**). The maps were generated using the *Google Maps API* and *Map Data ©2014 Google*.

To highlight why a value of *α* < 1 might be imperative to consider, in Fig. 5 we display two transportation networks whose operative airport (AN) connectivity is indistinguishable under two different attacks but that of their inoperative airports (IN) is drastically different. Since the largest cluster size in the Active Network under both attacks is the same, *S*_*A*_ = 167 airports, the standard metric of robustness (*α* = 1) would rank them equally robust (see also Fig. 5c). However, the largest cluster size in the IN of Fig. 5a (attack 1) is more than 6 times smaller compared to the one shown in Fig. 5b (attack 2). This results in *R*_*N*_(*α* = 0) ≈ 1 for attack 1 and *R*_*N*_(*α* = 0) ≈ 0 for attack 2 (See Fig. 5c). It is obvious that these two scenarios are significantly different in view of economic, logistic, security, and other aspects. Consider for instance the monetary losses, conceptually approximated by the amount of traffic lost due to removal of airports (temporarily due to natural hazards, or permanently due to structural airline reorganization). We roughly approximate the lost traffic by the number of lost edges in the network, and use the largest cluster size in IN as a proxy for this quantity. Naturally, losing a certain number of interconnected airports leads to more severe traffic losses than losing the same number of disconnected airports. Incorporating more realistic scenarios, by including a gradual recovery of affected airports, demands that the connectivity of the idle airports must be considered to correctly assess network robustness. Thus, the reactivation of a disconnected airport in the Idle Network would lead to a restoration of its total functionality, while if the affected airport belongs to a cluster of formerly connected airports (Idle Network), its activation would not signify a recovery of its functionality, which would remain diminished until all its connections are brought back online as well (e.g., consider as an extreme case a recovered airport, where all its connections remain offline).

Furthermore, we highlight the prospective use of this framework to detect critical nodes in networks. Critical nodes are defined as nodes whose removal is detrimental for the overall network connectivity^45–49^, as for example, nodes whose removal results in the largest decrease of the pairwise connectivity among the remaining nodes. However, currently the assessment of critical nodes is not informed by the change in the IN connectivity under an attack. Thus, we argue that critical nodes should be assessed by considering both the effect of their removal from the AN and the implication of this removal on the IN, and thereby defining a general functional that properly weighs both effects similar to the one used to define network robustness in Eqs. 4 and 5.

Networks are ubiquitous in natural, social and human-designed systems. The robustness of those systems, i.e., their capacity to maintain functionality, is a fundamental property that is critical to be correctly assessed, especially in the case wherein the system is subject to stressors that can trigger cascading failures. In this paper, we argue that a critical property–the connectivity of the affected nodes (Idle Network)–has been consistently neglected in network robustness analyses, leading to potentially misleading assessments. We have shown that in general the information content of the connectivity of the idle nodes is not redundant with respect to the connectivity in the Active Network. We illustrate the dual perspective framework of robustness assessment by introducing a simple (linear) generalization of robustness, and showing its significant quantitative and qualitative implications, such as re-ranking of network robustness under different attacks. Finally, we emphasize the potential of this framework for prospective studies where recovery processes acting on the affected nodes (Idle Network) are implemented. In those cases, the underlying connectivity in-between the Active and Idle Networks might also be revealed as important components for recovery rate assessments.

## Methods

We propose a dual perspective approach in assessing the robustness of networks wherein at any instant in the network evolution under attack, two distinct networks are defined: (i) the Active Network (AN) composed of the unaffected nodes and (ii) the Idle Network (IN) composed of the affected nodes. We test the validity of our framework by analyzing three prototype networks and an airline network under sequential node removal.

### Prototype networks

Three prototype networks are used: (i) a *square lattice* consisting of *T* = 10,000 nodes arranged in a Von Neumann neighborhood (i.e., each node having four neighbors); (ii) *Tokunaga self-similar trees* ^34^ (T-tree), which are known to describe critical binary Galton-Watson processes^50^ and level-set tree representation of symmetric random walks or regular Brownian motion^51^. Tokunaga trees with a broad range of parameter values have found wide applicability in describing the topology of river networks^34, 35, 52^, biological networks (leaves and cardiovascular systems)^53^ and clustering of earthquake aftershocks^54^. In this paper, we use Tokunaga trees of order Ω = 6, where each Horton-Strahler branch^7, 35^ in the tree represents a node, and parameters (*a*, *c*) = (1, 2); (iii) and Barabasi-Albert (BA) scale-free network^2^, a system with heterogeneous node degree distribution that exhibits high connectivity and contains intricate structures due to the presence of loops. The BA network incorporates preferential attachment and growth mechanisms. We construct a BA network using an initially connected network of *m*_*0*_ = 3 nodes and adding a new node with *m* = 2 links per time step, until *T* = 1,000 nodes are added.

### Airline network

We also analyzed a real-world network corresponding to the flight connections of the second largest European airline, RyanAir^44^ as of February 17, 2014. The examined network consists of 186 airports and 1,507 edges that represent the existence of at least one weekly flight between the respective airports. The maps used in the network illustrations were generated using the *Google Maps API* and *Map Data ©2014 Google*.

### Sequential node removal

We examined three different strategies of node elimination, where at each time step one node is removed from the Active Network: (i) Random Failure (RF) nodes are removed at random using a discrete uniform distribution over all the active nodes; (ii) Targeted Attack (TA) assigns a removal probability to each vertex proportional to its initial degree of connectivity in the Active Network; and (iii) Random Spreading (RS) where the first node is removed at random as in RF, and afterwards, at each time step one node connected to an eliminated node is randomly removed.

## Electronic supplementary material

Supplementary Material

## References

[1] Watts DJ, Strogatz SH (1998). Collective dynamics of ‘small-world’ networks. Nature.

[2] Barabasi A-L, Albert R (1999). Emergence of scaling in random networks. Science.

[3] Newman, M. E. J. *Networks: An Introduction* pp. 784 (Oxford University Press, 2010).

[4] Boccaletti S, Latora V, Moreno Y, Chavez M, Hwang D-U (2006). Complex networks: Structure and dynamics. Phys. Rep..

[5] Schneider CM, Moreira AA, Andrade JS, Havlin S, Herrmann HJ (2011). Mitigation of malicious attacks on networks. Proc. Natl. Acad. Sci..

[6] Barrat, A., Barthelemy, M. & Vespignani, A. *Dynamical Processes On Complex Networks*. (Cambridge University Press, 2008).

[7] Rodriguez-Iturbe, I. & Rinaldo, A. *Fractal River Basins: Chance and Self-Organization*. (Cambridge University Press, 1997).

[8] Tejedor A, Longjas A, Zaliapin I, Foufoula-Georgiou E (2015). Delta channel networks: 1. A graph-theoretic approach for studying connectivity and steady state transport on deltaic surfaces. Water Resour. Res..

[9] Tejedor A, Longjas A, Zaliapin I, Foufoula-Georgiou E (2015). Delta channel networks: 2. Metrics of topologic and dynamic complexity for delta comparison, physical inference, and vulnerability assessment. Water Resour. Res..

[10] Tejedor A (2016). Quantifying the signature of sediment composition on the topologic and dynamic complexity of river delta channel networks and inferences toward delta classification. Geophys. Res. Lett..

[11] Albert R, Jeong H, Barabasi A-L (2000). Error and attack tolerance of complex networks. Nature.

[12] Cohen R, Erez K, ben-Avraham D, Havlin S (2000). Resilience of the Internet to random breakdowns. Phys. Rev. Lett..

[13] Callaway DS, Newman MEJ, Strogatz SH, Watts DJ (2000). Network robustness and fragility: percolation on random graphs. Phys. Rev. Lett..

[14] Cohen R, Erez K, ben-Avraham D, Havlin S (2001). Breakdown of the Internet under intentional attack. Phys. Rev. Lett..

[15] Holme P, Kim BJ, Yoon CN, Han SK (2002). Attack vulnerability of complex networks. Phys. Rev. E.

[16] Albert R, Barabasi A-L (2002). Statistical mechanics of complex networks. Rev. Mod. Phys..

[17] Motter AE, Lai Y (2002). Cascade-based attacks on complex networks. Phys. Rev. E.

[18] Shargel B, Sayama H, Epstein I, Bar-Yam Y (2003). Optimization of robustness and connectivity in complex networks. Phys. Rev. Lett..

[19] Vázquez A, Flammini A, Maritan A, Vespignani A (2003). Modeling of Protein Interaction Networks. ComPlexUs.

[20] Song C, Havlin S, Makse HA (2006). Origins of fractality in the growth of complex networks. Nat. Phys..

[21] Buldyrev SV, Parshani R, Paul G, Stanley HE, Havlin S (2010). Catastrophic cascade of failures in interdependent networks. Nature.

[22] Parshani R, Buldyrev SV, Havlin S (2011). Critical effect of dependency groups on the function of networks. Proc. Natl. Acad. Sci. USA.

[23] Gallos LK, Barttfeld P, Havlin S, Sigman M, Makse HA (2012). Collective behavior in the spatial spreading of obesity. Sci. Rep..

[24] Iyer S, Killingback T, Sundaram B, Wang Z (2013). Attack robustness and centrality of complex networks. PloS ONE.

[25] Schneider CM, Yazdani N, Aráujo NAM, Havlin S, Herrmann HJ (2013). Towards designing robust coupled networks. Sci. Rep..

[26] Verma T, Araújo NAM, Herrmann HJ (2014). Revealing the structure of the world airline network. Sci. Rep..

[27] Daqing L, Yinan J, Rui K, Havlin S (2014). Spatial correlation analysis of cascading failures: Congestions and Blackouts. Sci. Rep..

[28] Wang J, Xu B, Wu Y (2015). Ability paradox of cascading model based on betweenness. Sci. Rep..

[29] Gong M, Ma L, Cai Q, Jiao L (2015). Enhancing robustness of coupled networks under targeted recoveries. Sci. Rep..

[30] Bassett DS, Bullmore E (2016). Small-World Brain Networks. The Neuroscientist.

[31] Strayer DL (2004). Changing Perspectives on Pearly Mussels, North America's Most Imperiled Animals. BioScience.

[32] Wasserman, S. & Faust, K. *Social Networks Analysis* (Cambridge University Press, 1994).

[33] Kosterev DN, Taylor CW, Mittlestadt WA (1999). Model Validation of the August 10, 1996 WSCC System Outage. IEEE T. Power Syst..

[34] Zaliapin I, Foufoula-Georgiou E, Ghil M (2010). Transport on river networks: A dynamic tree approach. J. Geophys. Res. Earth Surf..

[35] Peckham S (1995). New results for self-similar trees with applications to river networks. Water Resour. Res..

[36] Jeong H, Tombor B, Albert R, Oltvai ZN, Barabasi A-L (2000). The large-scale organization of metabolic networks. Nature.

[37] Latora V, Marchiori M (2001). Efficient Behavior of Small-World Networks. Phys. Rev. Lett..

[38] Latora V, Marchiori M (2003). Economic small-world behavior in weighted networks. Eur. Phys. J. B.

[39] Crucitti P, Latora V, Marchiori M, Rapisarda A (2003). Efficiency of scale-free networks: error and attack tolerance. Physica A.

[40] Crucitti P, Latora V, Marchiori M, Rapisarda A (2004). Error and attack tolerance of complex networks. Physica A.

[41] Holme P (2002). Edge overload breakdown in evolving networks. Phys. Rev. E.

[42] Friedel CC, Zimmer R (2007). Influence of degree correlations on network structure and stability in protein-protein interaction networks. BMC Bioinformatics.

[43] Magoni D (2003). Tearing down the Internet. IEEE J. Sel. Area. Comm..

[44] *Data Source*: Ryanair Airlines, http://www.ryanair.com/, Accessed: 17/02/2014.

[45] Arulselvan A, Commander CW, Elefteriadou L, Pardalos PM (2009). Detecting critical nodes in sparse graphs. Comput. Oper. Res..

[46] Arulselvan, A., Commander, C., Shylo, O. & Pardalos, P. Cardinality-constrained critical node detection problem. In: *Performance Models and Risk Management in Communications Systems*, Gulpinar, N., Harrison, P. & Rustem, B. (eds) 79–91 (Springer, 2011).

[47] Shen Y, Nguyen NP, Xuan Y, Thai MT (2013). On the discovery of critical links and nodes for assessing network vulnerability. IEEE/ACM Trans. Netw..

[48] Kuhlman, C. J., Anil Kumar, V. S., Marathe, M. V., Ravi, S. S. & Rosenkrantz, D. J. Finding Critical Nodes for Inhibiting Diffusion of Complex Contagions in Social Networks. In: *Machine Learning and Knowledge Discovery in Databases*. Balcázar, J. L., Bonchi, F., Gionis, A., Sebag, M. (eds) (Springer, 2010).

[49] Borgatti S (2006). Identifying sets of key players in a social network. Comput. Math. Organiz. Theor..

[50] Burd GA, Waymire EC, Winn RD (2000). A self-similar invariance of critical binary Galton-Watson trees. Bernoulli.

[51] Zaliapin I, Kovchegov Y (2012). Tokunaga and Horton self-similarity for level set trees of Markov chains. Chaos Soliton. Fract..

[52] Zanardo S, Zaliapin I, Foufoula-Georgiou E (2013). Are American rivers Tokunaga self-similar? New results on river network topology and its climatic dependence. J. Geophys. Res. Earth Surf..

[53] Turcotte DL, Pelletier JD, Newman WI (1998). Networks with side branching in Biology. J. Theor. Biol..

[54] Turcotte DL, Holliday JR, Rundle JB (2007). BASS, an alternative to ETAS. Geophys. Res. Lett..

