# Supplementary material for: Network robustness assessed within a dual connectivity framework: joint dynamics of the Active and Idle Networks
Source: Sci Rep. 2017 Aug 17;7:8567. doi: 10.1038/s41598-017-08714-3 (PMC5561179; doi:10.1038/s41598-017-08714-3)
Supplement: Supplementary file 1 — Supplementary Material [file 41598_2017_8714_MOESM1_ESM.pdf]

Supplementary Material

Network robustness assessed within a dual connectivity framework: joint dynamics of the Active and Idle Networks

[Paper # SREP-17-04964]

Alejandro Tejedor, Anthony Longjas, Ilya Zaliapin, Samuel Ambroj and Efi Foufoula-Georgiou

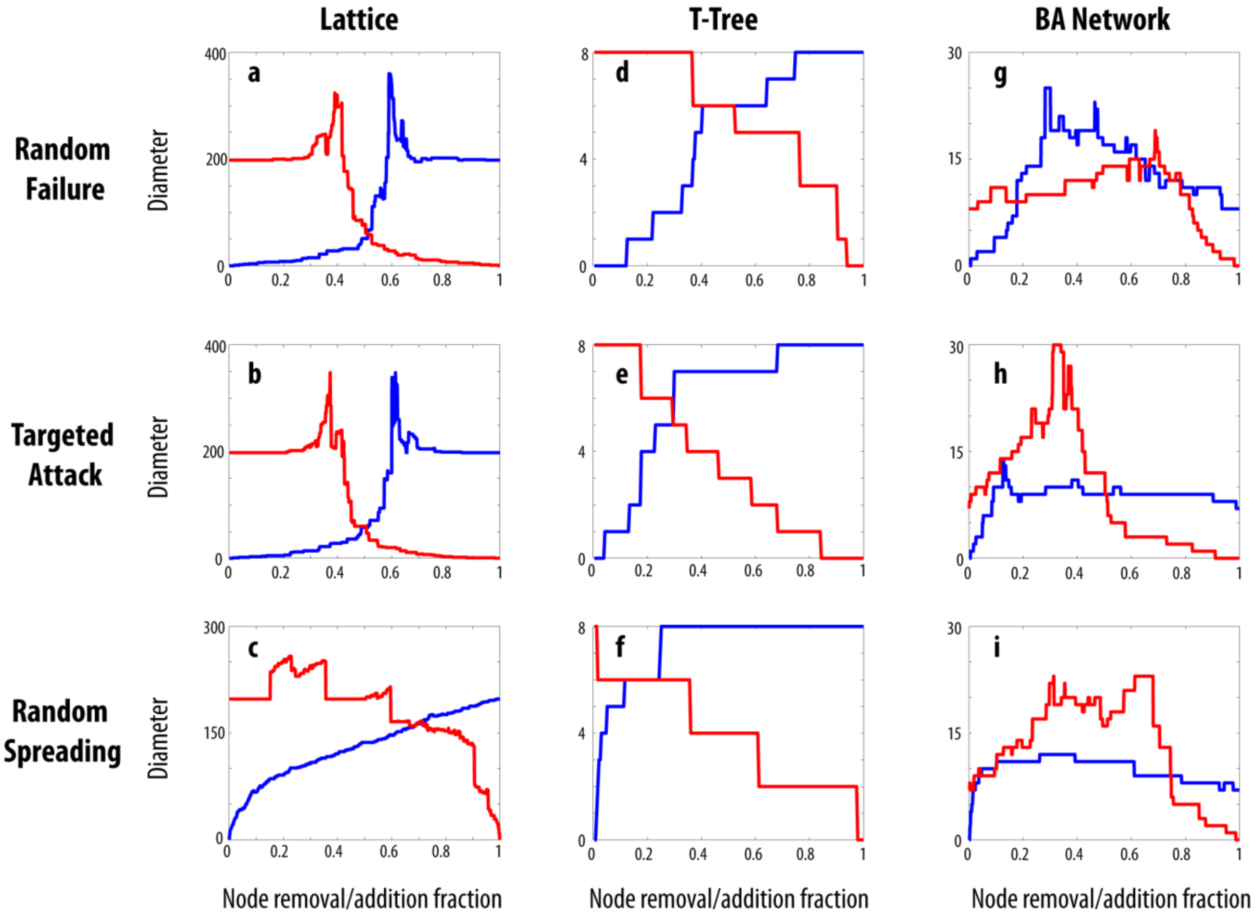

Figure S1 | Dual connectivity perspective evolution of networks under attack using the diameter as proxy for connectivity. Diameter in the Active Network, AN (red) and Idle Network, IN (blue) for the lattice, Tokunaga Tree and BA networks with respect to three different sequential node removal strategies: panels **a, d, g –** random failure, **b, e, h** – targeted attack, and **c**, **f**, **i** – random spreading. The time are normalized by the system size. The diameter at time *t* is computed as the longest distance (measured in terms of number of edges) in between any pair of nodes within the network, given that both nodes belong to the same component (cluster). The lack of a general symmetry, even complementariness, revealed by the temporal evolution of the largest cluster size in both AN and IN (see Fig. 2) are confirmed by the evolution of the diameter highlighting the necessity to monitor both networks (AN and IN) to assess the overall network robustness.

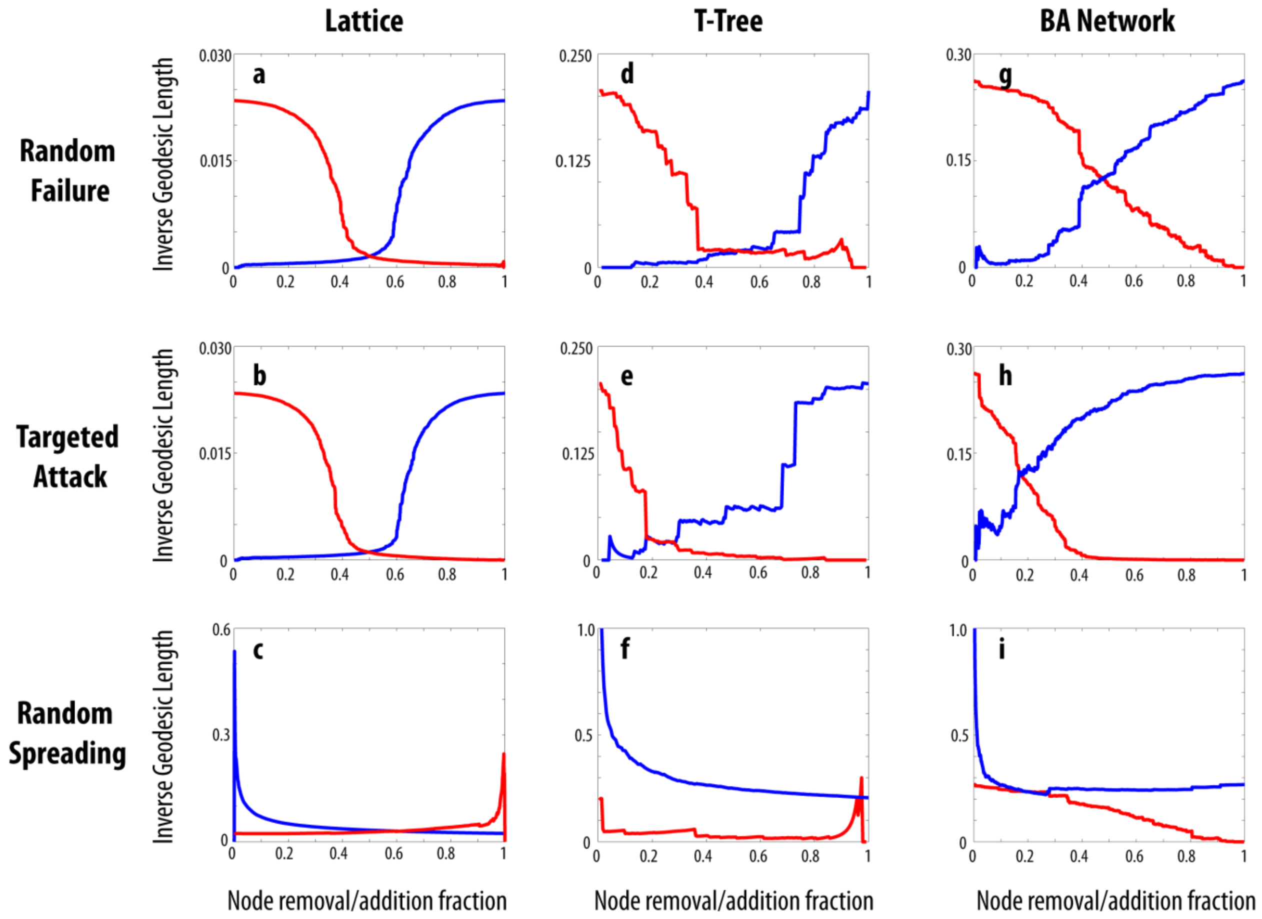

Figure S2 | Dual connectivity perspective evolution of networks under attack using inverse geodesic length as proxy for connectivity. Evolution of inverse geodesic length in the Active Network, AN (red) and Idle Network, IN (blue) for the lattice, Tokunaga Tree and BA networks with respect to three different sequential node removal strategies: panels **a, d, g –** random failure, **b, e, h** – targeted attack, and **c**, **f**, **i** – random spreading. The time are normalized by the system size. The average inverse geodesic length is computed as: $l^{-1} = \langle \frac{1}{d(n_i, n_j)} \rangle = \frac{1}{T(T-1)} \sum_{i=1}^{T} \sum_{j=1, j \neq i}^{T} \frac{1}{d(n_i, n_j)}$, where T is the number of nodes, and $d(n_i, n_j)$ is the geodesic length between nodes n_i and n_j, *i.e.* the number of edges forming the shortest path that connects nodes n_i and n_j. The lack of a general symmetry, even complementariness, revealed by the temporal evolution of the largest cluster size in both AN and IN (see Fig. 2) are confirmed by the evolution of the average inverse geodesic length highlighting the necessity to monitor both networks (AN and IN) to assess the overall network robustness.

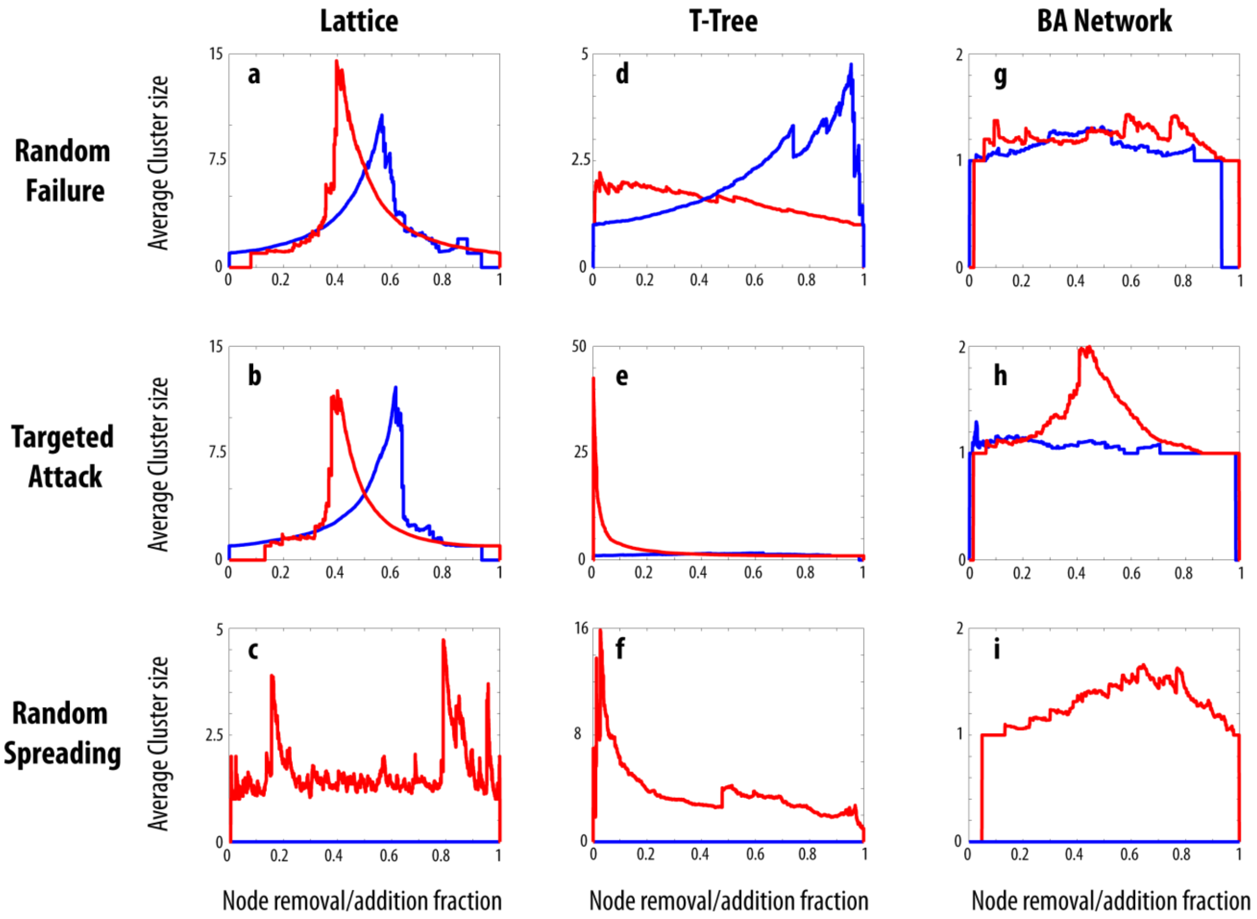

Figure S3 | Dual connectivity perspective evolution of networks under attack using average cluster size as proxy for connectivity. Evolution of the average cluster size in the Active Network, AN (red) and Idle Network, IN (blue) for the lattice, Tokunaga Tree and BA networks with respect to three different sequential node removal strategies: panels **a, d, g –** random failure, **b, e, h** – targeted attack, and **c**, **f**, **i** – random spreading. The time are normalized by the system size. The average cluster size at time *t* is computed as the arithmetic mean of the sizes of all the disconnected components (clusters) when the largest component is excluded. Note that given the nature of the random spreading attack and the definition of this metric, the average cluster size is zero for the IN for the three networks analyzed. The lack of a general symmetry, even complementariness, revealed by the temporal evolution of the largest cluster size in both AN and IN (see Fig. 2) are confirmed by the evolution of the average cluster size highlighting the necessity to monitor both networks (AN and IN) to assess the overall network robustness.

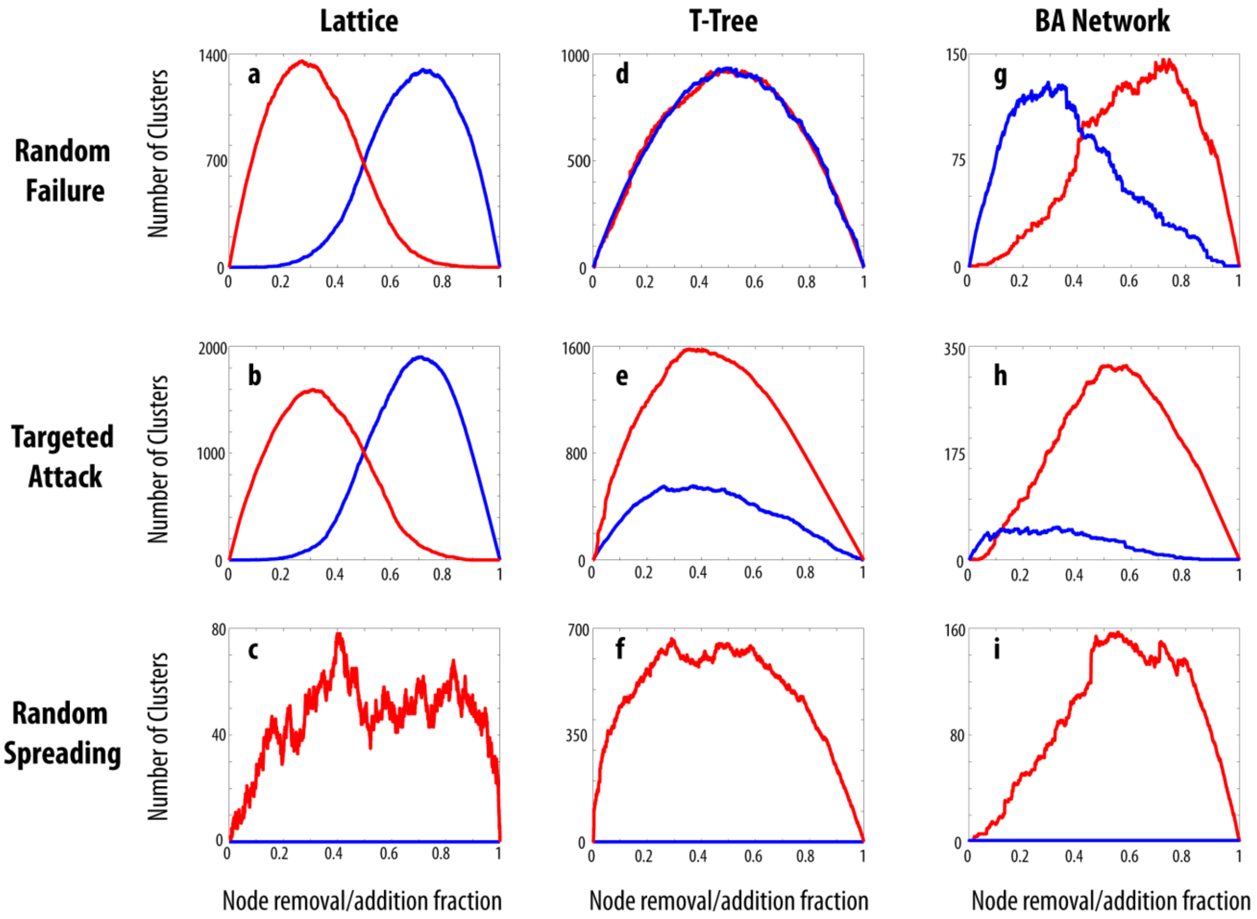

Figure S4 | Dual connectivity perspective evolution of networks under attack using number of clusters as proxy for connectivity. Evolution of number of clusters in the Active Network, AN (red) and Idle Network, IN (blue) for the lattice, Tokunaga Tree and BA networks with respect to three different sequential node removal strategies: panels **a, d, g –** random failure, **b, e, h** – targeted attack, and **c**, **f**, **i** – random spreading. The time are normalized by the system size. The number of clusters at time *t* is simply computed as the count of disconnected components (clusters) at that stage of the attack. Note that given the nature of the Random spreading attack and the definition of this metric, the number of clusters is equal to one for the IN for the three networks analyzed. The lack of a general symmetry, even complementariness, revealed by the temporal evolution of the largest cluster size in both AN and IN (see Fig. 2) are confirmed by the evolution of the number of clusters highlighting the necessity to monitor both networks (AN and IN) to assess the overall network robustness.
